# Progress in Gynæcology

**Published:** 1902-02-08

**Authors:** 


### PROGRESS IN GYNÆCOLOGY.

**Cancer of the Uterus.**—Dr. F. J. McCann,[1] in an instructive paper on the diagnosis of cancer of the uterus, draws attention to the fact that there still exists a widespread belief that the great sign of cancer of the womb is the presence of a putrid discharge, whereas this often does not occur until the disease has made considerable headway, and indeed indicates that the cancerous mass is beginning to slough. Pain is also another of the later symptoms, and does not occur until the disease has spread to the pelvic peritoneum and cellular tissue, and if at all severe usually means that the disease has advanced too far for eradication. In cancer of the body of the uterus pain is stated to be an earlier symptom than in cervical cancer, but McCann believes that even in these cases the disease advances considerably before causing any painful sensations. Dr. Kynock,[2] writing on the surgical treatment of cancer of the uterus remarks that at the recent congress in Berlin and in Giessen it was shown that the results of operation in cancer of the uterus compared very favourably with those gained by operation for cancer of the breast or rectum. In discussing the relative advantages of operation by the abdominal and vaginal routes, it was pointed out that the pelvic glands are comparatively late in being affected, as compared with the paravaginal and parametric tissue, and extirpation of the pelvic glands by the abdominal route was not favoured. The general indications for operation were summed up as folows : (1) In all cases where carcinoma is limited to the uterus the vaginal route is to be preferred ; (2) considering the advantages of the vaginal and dangers of the abdominal operation the indications for the former should be extended ; (3) the abdominal operation should only be undertaken when the cancer is complicated with myoma or ovarian cyst.

**The Treatment of Uterine Fibroids.**—When and how to operate on fibroids was the subject of an interesting discussion at the annual meeting of the British Medical Association. The discussion was opened by Dr. Duncan,[3] who pointed out that, although the direct mortality from fibroids was a matter of controversy, the danger to health was generally admitted, and was far more important as regards the general question of operation. Although there is no doubt that many cases give no trouble and should be left alone, others so seriously affect the health of the patient that the question of operation arises. Dr. Duncan considers that the indications for operation are (1) hæmorrhage ; (2) pressure symptoms ; (3) rapid increase in size ; (4) complication with pregnancy. In cases of hæmorrhage he advocated that, in the first place, the cervix should be dilated, in order to remove any polypus that might be present ; but if the tumour turned out to be interstitial, he thought it was bad surgery to incise the capsule and give ergot in the hope of expulsion, and he equally deprecated removal by morcellement. He considered that oöphorectomy had very properly fallen into disuse, and he had found no benefit from Apostoli's treatment. Myomectomy was no doubt a good way of dealing with subperitoneal growths, if single and not widely attached. The choice of operation finally lay between retro-peritoneal hysterectomy and pan-hysterectomy, and of these he preferred the former. (With regard to vaginal hysterectomy he thought that it had special drawbacks and risks that were not compensated by advantages.) He secured the ovarian and uterine vessels before cutting the peritoneal flaps, so that there was a minimum of hæmorrhage; he did not sew up the stump itself, but secured the peritoneum over it. Mr. Alban Doran[4] thought that if there was any doubt as to the position of the uterine arteries it was better to amputate the uterus and to secure the arteries as they spouted. Christopher Martin[5] considered that in most cases where operation was necessary hysterectomy would be required, and he did not think there was much to choose between pan-hysterectomy and supra-vaginal amputation.

**Intraligamentous Cysts.**—C. D. Palmer,[6] in a paper on this subject, maintains that all kinds of cysts, whether oöphoritic, paroöphoritic, or parovarian, may grow into the folds of the broad ligament and become intraligamentous, the paroöphoritic being most apt to take on this special course of movement in their progressive development. He points out that the removal of these intraligamentous cysts demands the possession of superior skill, together with a correct knowledge of the parts and surroundings. These cysts are, as a rule, easily peeled out of the broad ligaments. Dr. Palmer refers to the danger of wounding the ureters or the uterine arteries, and earnestly advocates the advisability of completely exsecting the uterus as well as the appendages, when the cysts are bilateral or when they are deeply embedded in surrounding textures.

**Leucoplakia of the Vulva and Cancer.**—Butlin[7] records three interesting cases of leucoplakia of the vulva associated with epithelioma. The plaques were present on the mucous surface only and not on the skin. They resembled in appearance, feel, and variety of form, the white plaques which are met with upon the mucous surface of the mouth; several cases have been recorded in which the vulva and mouth have been attacked in the same patient, and there is reason to believe that it is the same disease in both situations. In neither of Butlin's cases was there any reason to suspect the existence of syphilis, inherited or acquired, and he is inclined to regard the condition as a degeneration rather than an inflammation. He draws attention to the predisposition of this altered surface to the growth of cancer, and thinks that the question arises as to the desirability of the free removal of such plaques from the vulva, whether the signs of development of cancer are present or not.

**Ectopic Gestation.**—Alban Doran[8] reports an instructive case of tubal gestation in which the sac was entirely anterior to the uterus. Although the position of the swelling was very exceptional, the other symptoms of tubal gestation were well marked. An oval mass of the size of a turkey's egg lay in front of the uterus, pushing it backwards. The abdomen was opened and the tumour found to consist of the dilated left tube bent round to the right, the vermiform appendix being adherent to it. The dilated tube was removed and the appendix amputated close to the caecum. On examining the specimen the middle part of the tube was found to be greatly dilated and contained blood clot with well marked chorionic villi. The ovary was small, and contained a recent corpus luteum. G. E. Shoemaker[9] gives the details of three cases of suppurating hæmatocele due to extra-uterine pregnancy. In neither of the cases was there any history of a missed period, and none of the patients had actual collapse. Operation in each case was undertaken by the abdominal route, and Dr. Shoemaker remarks that vaginal incision and drainage is only adapted to a very few cases which have been thoroughly walled in and shut off from the general peritoneal cavity. Fritsch,[10] on the other hand, maintains that in the presence of an old, ruptured, tubal pregnancy which has formed a hæmatocele, the vaginal route should be adopted. It is not necessary to remove the ruptured tube, provided that all the contents of the sac are evacuated and free drainage is maintained. In recent tubal pregnancies, if the tumour exceeds the volume of a pear and is not plainly to be felt in Douglas's pouch, the abdominal route is, he thinks, preferable. H. Tuholske[11] reports a unique case of extra-uterine pregnancy. The ovum commenced to develop in the right Fallopian tube; tubal abortion occurred with complete extrusion of the unruptured gestation sac into the peritoneal cavity where the fœtus continued to develop in a most unusual situation. Probably as the result of hæmorrhage and position the sac was carried up to the diaphragm between the right lobe of the liver and the upper end of the kidney. In this position the establishment of placental connections allowed development to proceed until nearly full term. Operation was undertaken and a well-developed female child weighing 6½ lbs. removed. The patient died 32 hours after the operation, and at the post-mortem examination the diagnosis was confirmed. Histological examination showed original implantation in the ampulla of the right tube, and the formation of the placenta by the efficient transformation of the peritoneum and adjacent liver tissue. This case gives an answer to the question as to whether a fœtus, with its sac loosened from its original site and forming new connections might live to full term. Varnier and Sens[12] have collected and examined the records of sixty-five cases in which it seemed probable that ectopic gestation had occurred more than once in the same patient. They believe this to be more frequent than is generally supposed. As a rule ectopic gestation recurs in the tube of the opposite side. In the cases they studied it recurred but once in the same tube. In six cases of the 65 an intra-uterine pregnancy intervened between the ectopic gestations.

**The Surgical Treatment of Retro-displacement of the Uterus.**—F. B. Martin[13] recommends the following operation for simple retroversion of the uterus if free from adhesions. The ordinary Alexander incision is made on both sides. A pair of forceps is passed from one side under the skin and superficial fascia, and after grasping the ligament of the other side it is drawn through to the opposite side. The two ligaments are then tied in a knot, the uterus having previously been brought forward. He reports 61 cases with uniform success, and in six of these pregnancy occurred without any evil results.

Schücking,[14] after criticising the various operations for the cure of retro-displacement, advocates the following procedure: he opens the vesico-uterine pouch, and, after stretching or dividing any adhesions, brings the uterus forwards. He then passes a curved needle through the broad ligament just below the origin of the round ligament; this ligature is carried round the posterior aspect of the uterus, and made to emerge at a corresponding point in the left broad ligament. When this is tied the uterus is anteflexed. The surface at the angle of flexion is freshened with a curette. The peritoneum becomes adherent at this point, and thus maintains the uterus in its normal position, without impairing the mobility of the organ. Hivet[15] considers that intra-peritoneal shortening of the round ligaments is the most satisfactory operation for retroversion. He prefers this plan to Alexander's operation, because adhesions and morbid conditions of the appendages can be dealt with, and because the strongest portion of the round ligament, viz., the uterine portion, can be made use of for fixation. He thinks that no operative measures should be undertaken in the four following groups of cases: (1) Cases in which the displacement causes no symptoms; (2) displacements which can be kept reduced by a pessary; (3) displacements curable by massage; (4) displacements which form a part of a general visceral ptosis.

[1] Brit. Med. Jour., July 13, 1901. [2] Scottish Med. and Surg. Jour., Aug. 1901. [3] Brit. Med. Jour., Aug. 10, 1901. [4] Lancet, Aug. 17, 1901. [5] Ibid. [6] Med. Rec., June 22, 1901. [7] Brit. Med. Jour., July 13, 1901. [8] Lancet, Sept. 14, 1901. [9] Annals of Surg., July 1901. [10] Med. Chron., July 1901. [11] Amer. Gynæc. and Obstet. Jour., Dec. 1901. [12] Amer. Jour. of Med. Sci., July 1901. [13] Med. Rec., June 15, 1901. [14] Amer. Jour. of Med. Sci., Aug. 1901. [15] La Gynécologie, Aug. 1901.

